# Overexpression of Mitochondrial Ligases Reverses Rotenone-Induced Effects in a *Drosophila* Model of Parkinson’s Disease

**DOI:** 10.3389/fnins.2019.00094

**Published:** 2019-02-14

**Authors:** Bartosz Doktór, Milena Damulewicz, Elzbieta Pyza

**Affiliations:** Department of Cell Biology and Imaging, Institute of Zoology and Biomedical Research, Jagiellonian University, Kraków, Poland

**Keywords:** mul1, park, synapses, autophagy, apoptosis, neurodegeneration

## Abstract

Mul1 and Park are two major mitochondrial ligases responsible for mitophagy. Damaged mitochondria that cannot be removed are a source of an increased level of free radicals, which in turn can destructively affect other cell organelles as well as entire cells. One of the toxins that damages mitochondria is rotenone, a neurotoxin that after exposure displays symptoms typical of Parkinson’s disease. In the present study, we showed that overexpressing genes encoding mitochondrial ligases protects neurons during treatment with rotenone. *Drosophila* strains with overexpressed *mul1* or *park* show a significantly reduced degeneration of dopaminergic neurons, as well as normal motor activity during exposure to rotenone. In the nervous system, rotenone affected synaptic proteins, including Synapsin, Synaptotagmin and Disk Large1, as well as the structure of synaptic vesicles, while high levels of Mul1 or Park suppressed degenerative events at synapses. We concluded that increased levels of mitochondrial ligases are neuroprotective and could be considered in developing new therapies for Parkinson’s disease.

## Introduction

Mitochondria are “the powerhouses of the cell” playing a crucial role in the control of intracellular metabolism. The main processes in mitochondria are electron transport and proton pumping with the energetic steps (oxidative phosphorylation) that harness the energy as ATP. However, these processes are not without risk for cells, and the electron transport chain is sensitive to multiple external stressors. One of the functional disturbances within mitochondria is the stressed-induced massive production of reactive oxygen radicals e.g., the superoxide anion (O_2_^•-^). These dismutate toward hydrogen peroxide (H_2_O_2_), which can subsequently react to become hydroxyl radicals (HO^•^) that are most harmful and destructive to cells (Sena and Chandel, 2012; Kehrer and Klotz, 2015). Numerous proteins, such as ligases and mitochondrial kinases, are responsible for the control of mitochondrial stability and their biological maintenance, with the main function of mitophagy (Youle and Narendra, 2011). This process involves the removal of damaged, old, or improperly functioning mitochondria by autophagy (Cornelissen et al., 2018). Disrupting mitochondrial processes may lead to the development of many diseases such as Parkinson’s disease (PD), a neurodegenerative disease. Given their high energy demands, neurons are cells that are most sensitive to mitochondrial damage (Trevisan et al., 2018; Zilocchi et al., 2018).

Two important and well-described mitochondrial proteins which control the stability of mitochondria are the Mul1 and Park E3 ubiquitin ligases. These proteins are responsible for promoting mitophagy and maintaining mitochondrial integrity and fusion-fission processes in *Drosophila* (Kitada et al., 1998; Yun et al., 2014). Mul1 is also involved in SUMOylation. Mutations in the genes encoding Mul1 and Park in *Drosophila* lead to typical PD symptoms such as motor disorders, sleep problems and degeneration of dopaminergic neurons (Clark et al., 2006; Park et al., 2006; Yun et al., 2014; Gokcal et al., 2017). The above symptoms may also be caused by various neurotoxins, one of which is rotenone. The mechanism of its action is based on the disruption of electron transport in mitochondria. It inhibits the transport of electrons from iron-sulfur centers in complex I on ubiquinone (Lindahl and Öberg, 1961). As a result, it triggers mitochondrial damage by increasing oxidative stress, leading to neuronal death. However, cells can counteract these changes by enhancing the activity of antioxidative enzymes i.e., catalase, superoxide dismutase, heme oxygenase-1, or glutathione peroxidase. All these proteins protect cells from oxidative stress-mediated programmed cell death, or apoptosis (Silva and Coutinho, 2010).

Neurodegenerative diseases can be studied using animal models, including the fruit fly *Drosophila melanogaster*. The *Drosophila* genome carries homologs of most of the genes involved in the development of Parkinson’s disease, with the notable exception of α-synuclein (Nagoshi, 2018). In addition, current genetic tools and their short period of development, allows successful manipulation of its genome to be performed (Duffy, 2002). Symptoms typical of Parkinson’s disease, e.g., dopaminergic neuron degeneration and motor disorders, can be induced in *Drosophila* by various neurotoxins, such as rotenone, which has been used in the present study, and MPTP (1-methyl-4-phenyl-1,2,3,6-tetrahydropyridine). Both toxins induce symptoms typical of Parkinson’s disease via mechanisms linked to oxidative stress (Coulom and Birman, 2004; Abolaji et al., 2018).

In the present study, we examined whether the strong expression of two major mitochondrial ligases may protect flies exposed to rotenone, against developing symptoms typical of Parkinson’s disease. We found that overexpressing genes encoding Mul1 and Park in all neurons in the *Drosophila* brain inhibits degeneration of dopaminergic neurons and the motor disorders caused by rotenone. In addition, we found that rotenone affects the structure of synapses and the expression of synaptic proteins in the brain of flies, but when the levels of Mul1 and Park were increased in parallel, synapse structure and the normal level of synaptic proteins were restored.

## Materials and Methods

### Animals

The following strains were used for the experiments: Canton S (obtained from Bloomington Drosophila Stock Centre), *elav*-Gal4 (expressing the yeast transcription factor GAL4 under control of the *elav* promoter, obtained from Bloomington Drosophila Stock Centre), UAS*-park* (overexpressing *park* under UAS control, kindly provided by Dr. Alex Whitworth, University of Sheffield, United Kingdom) and UAS*-mul1* overexpressing *mul1* under UAS control, kindly donated by Dr. Ming Guo, Brain Research Institute, United States. Measured using qPCR in 7-days old males, the level of *park* (*elav*-GAL4 > UAS-*park*) and *mul1* (*elav*-GAL4 > UAS-*MUL1*) expression equaled 220 and 430%, of the control values, respectively. Flies were maintained on a standard yeast-cornmeal-agar medium at 25 ± 1°C, under a day/night cycle LD 12:12 (12 h of light and 12 h of darkness). For the experiments, adult flies were transferred for 7 days to flasks containing cotton soaked with a 10% sucrose (BioShop) solution with either DMSO (control) or 500 μM rotenone (Sigma) dissolved in DMSO and 10% sucrose (experimental flies).

### Climbing Assay to Test Negative Geotaxis

Males, 7 days old (*N* = 30), were transferred into an empty vial. After a short recovery, flies were gently tapped to the bottom of their vial and after 16 s individuals that climbed vertically beyond a 5-cm marked line were counted. The experiment was carried out in dim red light under constant conditions and was repeated three times.

### Locomotor Activity and Sleep Analysis

Seven-day old male flies (*N* = 32), were transferred to small glass tubes containing the sugar-agar food medium. Vials were located in DAMS monitors (Drosophila Activity Monitoring System, TriKinetics) and placed in an incubator (25°C). Monitors were equipped with infrared sensors, which automatically recorded activity of the flies inside their vials every 5 min. For the first 5 days, monitors were held in LD 12:12 (12 h of light and 12 h of darkness) conditions and in constant darkness (DD) for the next 6 days. Results from the second day of recording were analyzed to estimate the total activity and duration of sleep during the day and night using a Microsoft Excel plugin – BeFly (kindly donated by Dr. E. Green from the Department of Genetics, University of Leicester) (Rosato and Kyriacou, 2006) and Python 22^1^. Sleep in flies is defined as the time for which they do not change their position for at least 5 min. The experiment was repeated three times. In LD 12:12 and DD the rhythm of locomotor activity was analyzed, and its period was measured in DD.

### Whole Brain Immunohistochemistry

Seven-day old male flies were fixed in 4% paraformaldehyde in 0.2% PBT for 3 h at 4°C. Isolated brains were washed six times in PBS for 5 min each time. Next, they were incubated in 5% normal goat serum (NGS) and 0.5% bovine serum albumin (BSA) for 30 min at room temperature. Subsequently, brains were incubated overnight with mouse primary anti-Tyrosine Hydroxylase (1:1000, ImmunoStar) serum. After, brains were washed six times in 0.2% PBT for 5 min each and incubated overnight at 4°C with secondary goat anti-mouse Cy3-conjugated (1:500, Jackson ImmunoResearch Lab) antibodies. Finally, brains were washed four times in 0.2% PBT, twice in PBS and mounted in a Vectashield medium and examined with a Zeiss LSM780 Laser Scanning confocal Microscope.

### Western Blots

Seven-day old male flies (*N* = 30) were frozen in liquid nitrogen and decapitated. Heads were homogenized by sonication in 30 μl of Laemmli buffer with a protease inhibitor (Boehringer, Mannheim), left for 30 min at 4°C and frozen at -20°C. Homogenates were centrifuged at 13,200 rpm for 1 h at 4°C. Supernatants were collected and denatured at 85°C for 5 min. The total protein level was measured by a Quant-iT Protein Assay Kit and Qubit fluorometer (Invitrogen). Afterward, 20 μg of protein from each supernatant was subjected to electrophoresis (NuPAGE 4–12% bis-Tris gels, Invitrogen) at 165 V for 40 min and then blotted by electrotransfer onto a PVDF membrane (Invitrogen) at 30 V for 60 min. The membrane was blocked in 5% non-fat dry milk in PBS with 0.1% Tween20 (TBS) for 1 h at 4°C and incubated with primary antibodies; anti-α tubulin (1:20 000, Abcam), anti-Synaptotagmin (3H2 2D7, 1:2, Developmental Studies Hybridoma Bank), anti-Synapsin (3C11, dilution 1:1000, Developmental Studies Hybridoma Bank), anti-Sod1 (1:5000, abgent**),** anti-Atg5 (1:500, Abcam), anti-Disk large1 (4F3, 1:1000, Developmental Studies Hybridoma Bank) and anti-Dcp-1 (1:1000, Cell Signaling) in 1% BSA in 0.1% TBS overnight at 4°C. Next, the membrane was washed 5 times in 0.1% TBS for 10 min and incubated with the secondary antibody conjugated with HRP (1: 10 000, Abcam) in 1% BSA in 0.1% TBS for 1 h at room temperature. Afterward the incubation membrane was washed five times in 0.1% TBS and immunodetected with the ECL detection system (Perkin Elmer). Densitometric analysis of Western Blots was performed by ImageJ. The experiment was repeated three times.

### qPCR

Seven-day old male flies were decapitated and their heads were fixed in 100% ethanol for 2 h, and the brains were isolated. Total RNA was isolated using TriReagent (MRC Inc.). Total RNA (5 μg) was used for reverse transcription [High-Capacity cDNA Reverse Transcription Kit (ThermoFisher)] according to the manufacturer’s protocol. 1000 ng cDNA (diluted 1:10) was used for quantitative PCR. Each experiment was repeated three times. Expression of the following genes was examined using SYBR Green (ThermoFisher) and primers (Genoplast):

*rpl32*: F – TATGCTAAGCTGTCGCACAAATG,

R – GAACTTCTTGAATCCGGTGGGC

*dlg1*: F – ACCTGGAGAACGTAACGCAC,

R – ATGCACCTGACTTTGGCTCT

*synaptotagmin*: F – CTGAGTCCGGTCTTCAACGAG,

R – ACACGAGCGTCTTGTTCATGG

*synapsin*: F – ACCGGCATTCAGCAAGGAC,

R – CCCGGAAGTATTTGGACCAGT

*atg5*: F – CCGGAGCCTTTCTATCTGATGA,

R – CCTGGTGTTCGGCGCTTAT

*sod1*: F- GGACCGCACTTCAATCCGTA,

R – TTGACTTGCTCAGCTCGTGT

*park*: F – ATTTGCCGGTAAGGAACTAAGC,

R – AAGTGGCCGACTGGATTTTCT

*mul1*: F – GCTATTGGTGAACTGGAGTTGGA,

R – AGCTTGAGTATCGTCGTTGTCTT

The reaction was performed using a StepOnePlus Real-Time PCR System (ThermoFisher). Data were collected as raw CT values and analyzed using the 2-ΔΔCT method. Gene expression was normalized on an arbitrary scale with the control.

### Transmission Electron Microscopy (TEM)

Heads of 1-week old males were fixed in cacodyl-buffered PFA (2.5%) and glutaraldehyde (2%) primary fixative for 2 h. They were post-fixed for 1 h in OsO_4_ (2%) in veronal acetate buffer. Subsequently, the heads were dehydrated in an alcohol series followed by propylene oxide and then embedded in Poly/Bed 812 resin (Polysciences). Ultrathin sections (65 nm thick) of the first neuropil (lamina) of the optic lobe were cut and contrasted with uranyl acetate and lead citrate. Images of tetrad synapses in the lamina, as a convenient type of characteristic synaptic contact, were taken using a Jeol JEM 2100 HT TEM. The experiment was repeated three times. Ten images were taken per repetition.

### Statistics

Statistical analyses were performed using the GraphPad Prism 6. Data were examined for distribution normality, and statistical tests were chosen accordingly. The Wilcoxon–Mann–Whitney and Kruskal–Wallis tests were performed to assess differences in sleep, total activity and for climbing assay results. The results obtained from the Western blot, for qPCR data, and immunohistochemistry were analyzed using a one-way ANOVA and Tukey test.

## Results

### The Effect of *Mul1* or *Park* Overexpression on Motor Activity

**FIGURE 1:**
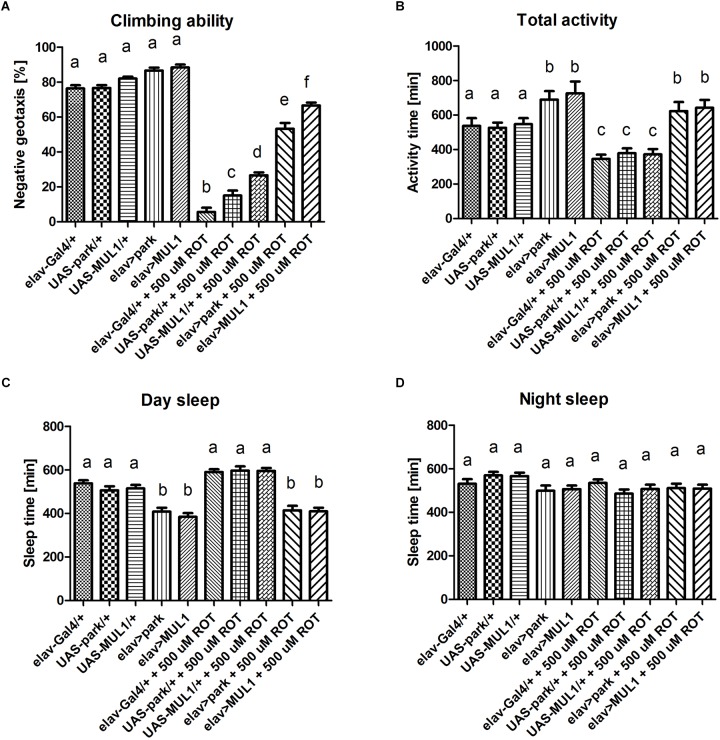
Overexpression of *mul1* or *park* restored motor activity. **(A)** *mul1* or *park* overexpression did not affect climbing ability in individuals who were not exposed to the toxin, but it significantly improved this form of activity in rotenone-treated subjects. Different letters (a,b,c) indicate statistically significant differences (*p* < 0.05, more precisely described in the Results section). **(B)** Flies with overexpressed *mul1* or *park* exhibited increased activity compared to control strains. These genetic modifications revealed prominent alterations in motor activity in rotenone-fed flies. Different letters (a,b,c) indicate statistically significant differences (*p* < 0.05, more precisely described in the Results section). **(C)** Individuals with increased Mul1 or Park level showed reduced sleep time during the day. Feeding flies with rotenone did not affect this form of behavior. Different letters (a,b,c) indicate statistically significant differences (*p* < 0.05, more precisely described in the Results section). **(D)** Rotenone and overexpression of *mul1* or *park* did not affect the sleep time at night in flies.

The analysis of *D. melanogaster* behavior revealed that overexpressing two major mitochondrial ligases in neurons increased the climbing ability and motor activity in flies treated with rotenone. Rotenone exposure reduced the climbing of flies by approximately 80% when compared with the control flies (*p* < 0.001) (Figure 1A). Overexpression of *mul1* or *park* in neurons of flies fed with rotenone, reversed this result and increased climbing by up to 70 and 60% in *elav* > *mul1* and *elav* > *park*, respectively, compared with the control strains (*elav*-Gal4 and UAS-*mul1* for *elav > mul1* and *elav*-Gal4 and UAS-*park* for *elav* > *park*) (*p* < 0.001). However, the overexpression of each of those two genes did not affect the climbing activity of untreated flies. Total activity during the day was also decreased after rotenone exposure by about 20%. Overexpression of *mul1* or *park* enhanced this activity in the control strains, and the differences between strains that overexpressed *mul1* or *park* were statistically significant relative to the controls (*p* < 0.05). It is worth noting that the overexpression of *mul1* or *park* restored the same activity level in experimental strains as in the control (*p* < 0.01) (Figure 1B). It should be pointed out that although the total activity was decreased, rotenone did not affect the length of sleep, neither during the day nor during the night (Figure 1C,D), while overexpressing *mul1* or *park* decreased sleep duration during the day, in both the experimental and control individuals by up to 20%, an increase that is statistically significant in control flies (*p* < 0.05) as well as in experimental strains (*p* < 0.01).

### The Effect of *Mul1* or *Park* Overexpression on Synaptic Proteins

**FIGURE 2:**
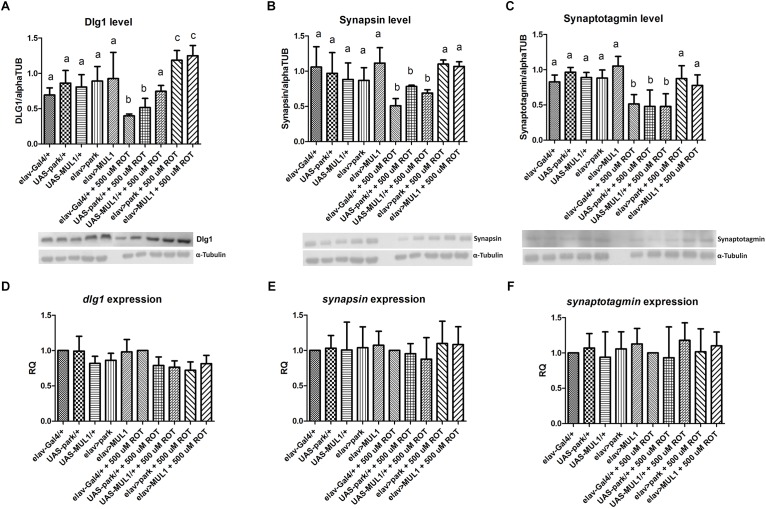
*mul1* or *park* overexpression increased the number of synaptic proteins reduced by rotenone. **(A–C)** *mul1* or *park* overexpression increased the level of individual synaptic proteins Disk Large1 **(A)**, Synapsin **(B)**, and Synaptotagmin **(C)** in the brain of rotenone-fed flies. Different letters (a,b,c) indicate statistically significant differences (*p* < 0.05, more precisely described in the Results section). **(D–F)** Overexpression of those two genes and rotenone did not affect the expression of genes encoded Disk Large1 **(D)**, Synapsin **(E)**, and Synaptotagmin **(F)**.

Analyses of the selected synaptic protein levels in the fly’s brain showed that rotenone reduces the abundance of these proteins, while overexpressing ligases restores their normal level in flies exposed to rotenone. Exposure to 500 μM rotenone reduced the level of Dlg1 (Figure 2A), Synapsin (Figure 2B) and Synaptotagmin (Figure 2C) to about 50% compared with the control. In the case of Dlg1 protein, statistically significant differences were found between *elav* > *mul1* and *elav*-Gal4 (*p* < 0.001), and UAS-*mul1* (*p* < 0.01), and also between *elav* > *park* and *elav*-Gal4, and UAS-*park* (*p* < 0.001) treated with rotenone. Differences between strains with overexpressed ligases in neurons and their respective controls were at *p* < 0.01 and *p* < 0.05, in the case of Synapsin and Synaptotagmin, respectively. The highest reduction of synaptic protein levels was observed in *elav*-Gal4/+ in all proteins examined. Overexpressing *mul1* or *park* did not affect the level of these proteins in the controls, but in all strains fed with the neurotoxin, the normal level was restored for Synapsin and Synaptotagmin while the Dlg1 protein level was about 20% higher than in the control. Both, rotenone and the overexpression of mitochondrial ligases did not affect expression of genes encoding the protein examined (Figure 2D–F).

### The Effect on Synapses of Rotenone and Overexpressing Mitochondrial Ligases

**FIGURE 3:**
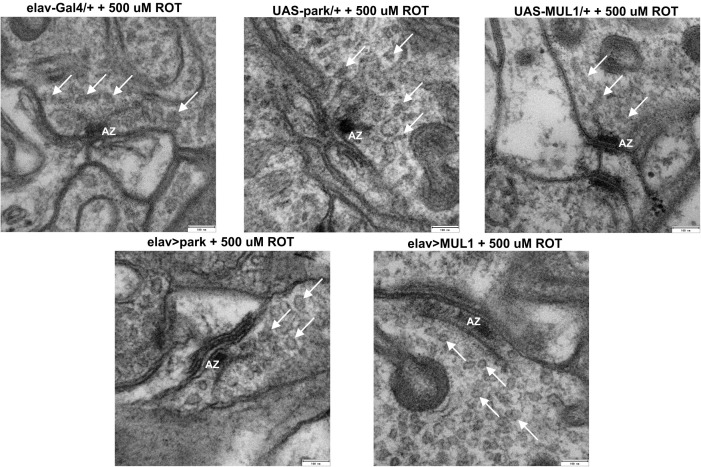
Overexpression of mitochondrial ligases restored correct morphology of active zones and synaptic vesicles. Flies fed with rotenone exhibited the altered shape of synaptic vesicles: they are elongated, less visible, with a slightly broken membrane and they are lighter. The “T-bar” activity zone is smaller, less visible, with almost absent platform. *mul1* or *park* overexpression restored the normal morphology of the synaptic vesicles in these flies: they are more round, full in the middle, and the active zone “T-bar” is larger, better visible, with a large platform Active Zone (AZ), White Arrow – synaptic vesicles).

TEM micrographs (Figure 3) of synapses examined in the lamina of the *Drosophila* visual system showed synapse distortion after exposure to rotenone. Among flies fed with rotenone, the presynaptic T-bar was smaller, and especially its platform, to which synaptic vesicles are attached, was smaller than in the control. Moreover, synaptic vesicles with irregular shapes were observed. They were more translucent than normal vesicles and their membrane was often broken. In strains with the overexpression of *mul1* or *park*, the active zone was large, with a clearly visible large T-bar platform, while synaptic vesicles were mostly round and electron dense, indicating they contained a transported cargo.

### The Effect of *Mul1* or *Park* Overexpression on Dopaminergic Neurons

**FIGURE 4:**
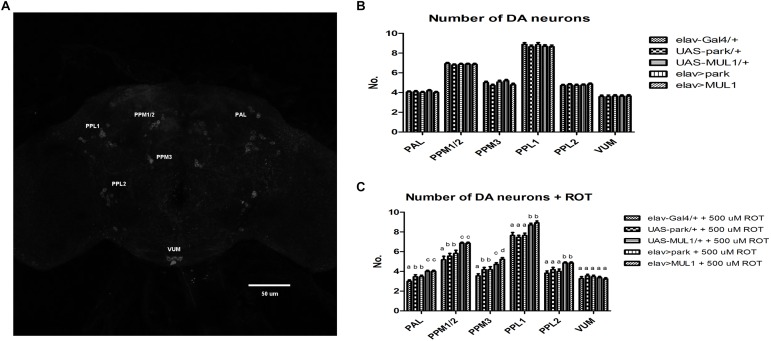
Overexpression of *mul1* or *park* restored the correct number of dopaminergic neurons. **(A)** An image of the *Drosophila* brain. Immunostaining with anti-tyrosine hydroxylase (anti-TH) antibodies. Six clusters of dopaminergic neurons are marked. **(B,C)** *mul1* or *park* overexpression did not change the number of dopaminergic neurons in flies’ brain **(B)**. However, it restores their correct number during feeding with rotenone **(C)** (*p* < 0.05, more precisely described in the Results section). Different letters indicate statistically significant differences.

Approximately 140 dopaminergic neurons have been described in six clusters per hemisphere in the brain of *Drosophila* by means of anti-TH antibodies (Nässel and Elekes, 1992; Pech et al., 2013); however, in the present study not all neurons from the PAM cluster were visualized (Figure 4A). Rotenone exposure caused degeneration of dopaminergic neurons in the five clusters that were examined, so that the number of these neurons was reduced by 23% in total (Figure 4C). In the case of the PAL, PPM1/2, and PPM3 clusters, the differences between strains with overexpressed ligases in their neurons and the corresponding *elav*-Gal4 control, were statistically significant at *p* < 0.01; between the same strains and their UAS controls the differences were significant at *p* < 0.05. In the PPL1 and PPL2 clusters, the differences between *elav* > *mul1* and *elav* > *park* and their controls were also statistically significant at *p* < 0.05. The increased level of Mul1 or Park in all neurons prevented neurodegeneration in toxin-fed individuals and the number of dopaminergic neurons was the same as in the control. The overexpression of *mul1* or *park* in the control strains did not change the number of dopaminergic cells in their brains (Figure 4B).

### The Effect of Overexpression of *Mul1* or *Park* and Rotenone on Autophagy and Apoptosis

**FIGURE 5:**
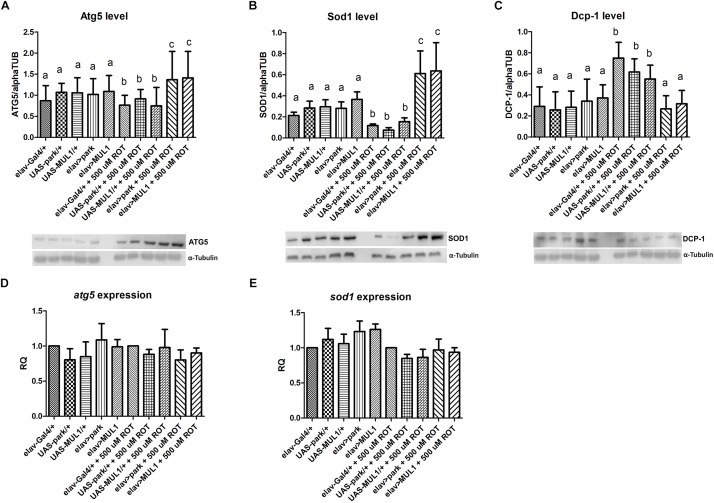
Effect of *mul1* or *park* overexpression on autophagy, apoptosis and antioxidant levels in flies fed with rotenone. **(A–C)** *mul1 or* *park* overexpression increased the level of Atg5 **(A)** and Sod1 (**B**) in flies fed with rotenone. This overexpression also decreased the level of Dcp-1 **(C)** in the toxin-fed flies but did not change the level of those proteins in control individuals. Different letters (a,b,c) indicate statistically significant differences (*p* < 0.05, more precisely described in the Results section). **(D,E)** Overexpression of mitochondrial ligases did not change the expression of genes encoding Atg5 and Sod1 proteins.

Rotenone also reduced the abundance of proteins involved in removing damaged organelles or entire cells from the body. It reduced the level of the Atg5 autophagy protein by about 30% (*p* < 0.05) when compared with all strains that were not fed the toxin, while the overexpression of *mul1* or *park* restored the normal level of Atg5 in rotenone-fed flies compared to Gal4 and UAS controls (*p* < 0.01) (Figure 5A). In the case of the protein Sod1, the profile was the same, while statistically significant differences were higher, between strains which overexpressed *mul1* or *park* compared with their rotenone-fed experimental groups (*p* < 0.001) (Figure 5B). Both rotenone and the increased level of Mul1 and Park did not change the expression of genes encoding these proteins (Figure 5D,E). Rotenone exposure also increased the Dcp-1 protein by about 100% in the brain of the fruit fly compared with Gal4 and UAS strains (*p* < 0.05) (Figure 5C). After overexpressing the ligases studied in rotenone-fed flies, the level of Dcp-1 was restored to the normal level, and this was also observed in individuals that were not treated with the toxin (*p* < 0.05).

## Discussion

Rotenone is a toxin that inhibits activity of the mitochondrial complex I and increases production of the radical oxygen species level, as shown in rats (Betarbet et al., 2000). In the present study, we showed that exposure to 500 μM rotenone in *Drosophila* (Coulom and Birman, 2004) reduces the levels of Atg5 and Sod1, which are important for cell survival under stress. Sod1 is an enzyme that is responsible for the elimination of free radicals from cells (Bunton-Stasyshyn et al., 2015). Its low-level leads to hypergeneration of reactive oxygen species in the cell and its presence triggers cellular damage (Henchcliffe and Beal, 2008). Atg5 is one of the major proteins involved in autophagy, during which damaged organelles can be removed and amino acid obtained for cell survival and homeostasis in mice (Kuma et al., 2004). The enhanced autophagy during high oxidative stress is beneficial for cells and delays the degenerative processes in rat cells (Chen et al., 2014). Its disruption is critical for PD pathogenesis in non-dopaminergic neurons and for the onset of non-motor symptoms in rats (Wise et al., 2018). The suppression of autophagy has an adverse effect on the elimination of free radicals (Navarro-Yepes et al., 2014) and we demonstrated that rotenone also affects apoptosis by increasing the level of Dcp-1 in fruit flies (Xu et al., 2009). It has already been observed that rotenone exposure induces changes in the level of several apoptotic proteins such as Bcl2, Bax, Caspase-8, and Cyt-C in rats (Dhanalakshmi et al., 2016) and that autophagy prevents oxidative stress-dependent apoptosis (Li et al., 2017).

Our results also showed that rotenone exposure disturbs climbing ability and locomotor activity of flies, during the day. This is most likely caused by increased free radical levels and apoptosis which have been observed in rats (Rosiñczuk et al., 2018), particularly in neurons, leading to neuronal disorders. It has been reported that oxidative stress is associated with behavioral disorders in mice (Camargo et al., 2018) and we demonstrated that the *mul1* or *park* overexpression in neurons can restore the normal level of apoptosis and increase autophagy and endogenous antioxidant enzyme levels. As a result, improvements in climbing and locomotor activity were observed. The results obtained also showed that overexpressing the ligases studied causes hyperactivity in flies and reduces their sleep time during the day. It has already been reported that increases in the level of mitophagy results in increased motor activity levels in fruit flies (Chakraborty et al., 2018) and this can explain hyperactivity in flies with higher levels of mitochondrial ligases. Mul1 and Park enhance mitochondrial fusion, so increasing their amount in neurons, most likely leads to the increased number of large mitochondria. In turn, large mitochondria produce more ATP than smaller ones (Sun et al., 2014), which in turn may increase the motor activity of *Drosophila*. Higher levels of Nix protein, a mitochondrial autophagy receptor, likewise increases ATP levels in strains that are also genetic models of Parkinson’s disease and shows that ATP levels depend on the quality of mitophagy (Koentjoro et al., 2017). Appropriate mitochondrial quality can also be provided by Afadin 6, an F-actin binding multidomain-containing scaffolding protein. This protein interacts with Park, as also reported in fruit flies, and its overexpression restores the physiological phenotype in the *pink* and *park* mutants (Basil et al., 2017). Our results suggest that the increased expression of *park* or *mul1* can also lead to an increase in the level of Afadin 6, which in turn is protective for mitochondria. Results obtained by other authors also showed that the *park* mutation in *Drosophila* decreases mass and cell size and increases sensitivity to oxygen radical stress (Pesah et al., 2004). The overexpression of *park* studied here may also increase cell size and reduce the cell sensitivity to free radicals, which was also a protective effect against rotenone. Results from other authors have shown that overexpressing the gene encoding Lrrk2, a leucine-rich repeat kinase, also involved in the development of PD, inhibits degeneration of dopaminergic neurons in the *Drosophila* model using rotenone (Ng et al., 2009). This inhibition suggests that overexpressing genes, the mutation of which cause symptoms of Parkinson’s disease, might be protective against sporadic forms of the disease in animal models exposed to neurotoxins. The Usp30 protein also appears to be associated with the development of Parkinson’s symptoms. This protein is located in mitochondria and acts as an inhibitor of mitophagy. It has been shown that overexpressing this protein removes ubiquitin attached by Parkin to damaged mitochondria and blocks the ability of Parkin to drive mitophagy (Bingol et al., 2014). We suggest that Usp30 activity is suppressed by the increased levels of Park and Mul1, which restore the correct mitophagy.

Behavioral disabilities may also result from damaged synapses in the nervous system and neuromotor junctions after exposure to rotenone. We observed that rotenone decreases the level of several proteins associated with synaptic transmission: Dlg1, Synapsin, and Synaptotagmin. Dlg1 is responsible for clustering neurotransmitter receptors and ion channels in the postsynaptic membrane and for mediating cell-cell adhesion (Kim et al., 2014). Synapsin is important for vesicle clustering in the presynaptic site and this protein also regulates synaptic plasticity (Vasin et al., 2014). In turn, Synaptotagmin acts as a Ca^2+^ sensor for fast neurotransmitter release (Geppert et al., 1994). Low levels of Synapsin or Synaptotagmin lead to poor synaptic transmission and motor disorders in rats (Sampedro-Piquero et al., 2014; Lai et al., 2015). Moreover, a low level of synaptic proteins is also correlated with a high level of free radicals, which as described above, reduce the amount of Sod1, increases apoptosis and decreases autophagy. However, the low level of a synaptic protein also results from the weak activity of translational factors of this protein translation, because of the action of free radicals. Lee et al. (Lee et al., 2018) have reported that a high level of ROS in human cells affects the activity of eIF2α translation factor. As shown by our results, rotenone decreases the level of proteins, synaptic proteins, Atg5 and Sod1, while the expression of their encoding genes remains normal. In order to confirm the lack of synaptic proteins affects, we conducted a TEM study of tetrad synapses in the first neuropil of the optic lobe of *D. melanogaster* as a model population of synapses. We found that rotenone intoxication affects the morphology of synaptic vesicles as well as the synaptic active zone, which are responsible for neurotransmitter exocytosis into the synaptic cleft. The active zone within the presynaptic T-bar is a site where Ca^2+^ triggered fusion of a synaptic vesicle occurs (Wichmann and Sigrist, 2010). The proper development of synapses depends on autophagy in *Drosophila* (Shen and Ganetzky, 2009) which is disrupted by rotenone as well as being affected by oxidative stress (Milton and Sweeney, 2012; Kamat et al., 2016). The observed changes in synapse morphology were not observed in strains with *mul1* and *park* overexpression. The T-bar active zones looked normal in both strains, T-bars were large with a large platform, and synaptic vesicles were round, dense in the middle, which is typical for well-functioning synapses.

Finally, we examined dopaminergic neurons after rotenone exposure in control and *mul1* or *park* overexpression strains. The results of other authors have shown that rotenone at a concentration of 500 μM causes degeneration of these neurons in five clusters: PAL, PPL1, PPL2, PPM1/2, and PPM3 (Coulom and Birman, 2004). These authors did not describe the VUM cluster, but the images included in their article show no reduction of the number of neurons in this group. This was also confirmed in another report (Navarro et al., 2014). The decrease of dopaminergic neuron numbers is associated with the reduction of the dopamine level, which is probably associated with motor disability (Smith et al., 2013; Santiago et al., 2014). After rotenone exposure we observed a decreased number of dopaminergic neurons in the five clusters of neurons previously mentioned, while the overexpression of mitochondrial ligases in neurons of strains fed with rotenone was protective against the toxin and prevented degeneration of the dopamine neurons. Given the association between dopaminergic neurons and motor activity, we propose that the inhibition of their degeneration probably results in the improved climbing ability of *Drosophila*.

To conclude, in the present study we showed that rotenone at a concentration of 500 μM reduces the level of autophagy, apoptosis and increases the level of free radicals. This leads to the disrupted function and morphology of synapses, locomotor activity and dopaminergic neurons in the brain of *D. melanogaster*. We also demonstrated that the overexpression of two main mitochondrial ligases: Mul1 and Park in all neurons is protective, since they inhibit the effect of rotenone. The degeneration of neurons seems to depend not only on the lack of ATP, because of the damaged mitochondria, but also because of their destructive effect in the cell.

## Author Contributions

EP was responsible for funding acquisition, conceptualized the study, administered the project, provided the resources, supervised the study, and also wrote, reviewed and edited the manuscript. BD performed data curation and formal analysis and visualized the study. BD and MD investigated the study and performed the methodology. MD and EP validated the study. BD, MD, and EP wrote the original draft of the manuscript.

## Conflict of Interest Statement

The authors declare that the research was conducted in the absence of any commercial or financial relationships that could be construed as a potential conflict of interest.

## Footnotes

1 http://www.python.org/
